# Elective laparoscopic cholecystectomy: recurrent biliary admissions predispose to difficult cholecystectomy

**DOI:** 10.1007/s00464-021-08986-x

**Published:** 2022-01-13

**Authors:** James Lucocq, John Scollay, Pradeep Patil

**Affiliations:** Department of General and Upper GI Surgery, Ninewells Hospital, University of Dundee, Dundee, UK

**Keywords:** Laparoscopic cholecystectomy, Admissions, Morbidity, Inflammation, Cholecystitis, Outcomes

## Abstract

**Introduction:**

Patients undergoing elective laparoscopic cholecystectomy (ELLC) represent a heterogeneous group making it challenging to stratify risk. The aim of this paper is to identify pre-operative factors associated with adverse peri- and post-operative outcomes in patients undergoing ELLC. This knowledge will help stratify risk, guide surgical decision making and better inform the consent process.

**Methods:**

All patients who underwent ELLC between January 2015 and December 2019 were included in the study. Pre-operative data and both peri- and post-operative outcomes were collected retrospectively from multiple databases using a deterministic records-linkage methodology. Patients were divided into groups based on clinical indication (i.e. biliary colic versus cholecystitis) and adverse outcomes were compared. Multivariate regression models were generated for each adverse outcome using pre-operative independent variables.

**Results:**

Two-thousand one hundred and sixty-six ELLC were identified. Rates of peri- and post-operative adverse outcomes were significantly higher in the cholecystitis versus biliary colic group and increased with number of admissions of cholecystitis (*p* < 0.05). Rates of subtotal (29.5%), intra-operative complication (9.8%), post-operative complications (19.6%), prolonged post-operative stay (45.9%) and re-admission (16.4%) were significant in the group of patients with ≥ 2 admissions with cholecystitis.

**Conclusion:**

Our data demonstrate that patients with repeated biliary admission (particularly cholecystitis) ultimately face an increased risk of a difficult ELLC with associated complications, prolonged post-operative stay and readmissions. These data provide robust evidence that individualised risk assessment and consent are necessary before ELLC. Strategies to minimise recurrent biliary admissions prior to LC should be implemented.

Laparoscopic cholecystectomy (LC) is performed in patients with a variety of different biliary pathologies, the majority of which include biliary colic, cholecystitis and choledocholithiasis/pancreatitis. Elective laparoscopic cholecystectomy (ELLC) is performed in symptomatic patients who have not required hospital admission or cases where a biliary issue has been managed non-operatively during one or more emergency hospital admissions.

When assessing a patients’ suitability for ELLC, it is important that surgeons have knowledge of risk factors associated with adverse peri-operative outcomes such as intra-operative and post-operative complications, prolonged post-operative stay and re-admission. This will help guide the decision to operate and form part of the informed consent process.

Patients undergoing cholecystectomy represent a heterogeneous group making it more challenging to stratify risk. Previous studies have identified factors associated with technically challenging surgery including the presence of acute cholecystitis, thickened gallbladder wall, comorbidities and age greater than 65 years [1–3]. Other studies have determined variables associated with post-operative complications following emergency LC [4].

Historic review of cholecystectomy data has often concentrated on rates of ductal injury [5]. However, more contemporary analysis has placed more emphasis on the generic complications experienced by patients undergoing biliary surgery, and it has been demonstrated that low grade morbidity is a relatively common occurrence following laparoscopic cholecystectomy [6].

The aim of this paper is to identify pre-operative factors associated with adverse peri- and post-operative outcomes in patients undergoing ELLC. This knowledge will help stratify risk, guide surgical decision making and better inform the consent process.

## Methods

### Population cohort

All patients who underwent ELLC between January 2015 and December 2019 were included in the study. This health board covers a defined geographical region with a stable population of approximately 493,000 people. Indications for ELLC included symptomatic biliary pathology (biliary colic, acute cholecystitis, choledocholithiasis/pancreatitis). Ethical approval was granted by the regional information governance committee. Patient written consent was not required.

### Data collection

Data were collected retrospectively from multiple databases using a deterministic records-linkage methodology. Patients were tracked between databases using a unique 10-digit patient identifier. These databases were used to obtain information relating to the index admission including baseline demographic and operative data. In addition, details of any significant complications (Clavien–Dindo classification ≥ 2) were recorded along with a description of any imaging or intervention performed in the post-operative period [7]. Total length of stay was recorded for all patients, and a prolonged post-operative stay was defined as one in which the patient stayed until at least the third post-operative day. Records of those patients who were readmitted under surgical care within 100 days of their operation were scrutinised for details of any further complications, imaging or intervention obtained. Unrelated elective and emergency admissions were excluded.

### Analysis

Patients were divided into groups based on clinical indication (i.e. biliary colic versus cholecystitis). Patients were assigned to the gallstone pancreatitis or choledocholithiasis group if the diagnosis was made without cholecystitis. Patients were then further divided by number of previous admissions. The rate of each adverse outcome was calculated for each group and compared using relative risk and chi-squared analysis.

Multivariate regression models were generated for each adverse outcome using pre-operative dependent variables. Variables included in the model included age (< 40; 40–59; ≥ 60 years), sex, ASA score (1; 2; ≥ 3), pre-operative ERCP (y/n), pre-operative cholecystostomy (y/n), main indication (biliary colic, cholecystitis, gallstone pancreatitis), ductal stones (y/n) and previous gallstone related admissions (1; 2; ≥ 3). The most parsimonious model for each adverse outcome was determined by eliminating insignificant variables using a top-down approach. All statistical tests were performed using the STATA/IC 16.1 software package.

## Results

Two-thousand one hundred and sixty-six ELLC were identified. The operations were performed under the care of 25 general surgical consultants. The surgical consultant was either the main operator or supervised a trainee in all cases.

Patients had a median age of 52 (range 13–92) years and 1579 were female (73%) (Table 1). Indication for surgery included biliary colic (58.1%), cholecystitis (32.5%), gallstone pancreatitis (4.2%), choledocholithiasis without gallstone pancreatitis (2.8%) and other less common indications (2.3%) such as biliary dyskinesia and polyps.

**Table 1:** Pre-operative data by indication for surgery

	Patient group				
	All patients*, (*n* = 2166)	Biliary colic, (*n* = 1262)	Cholecystitis, (*n* = 703)	Gallstone pancreatitis, (*n* = 91)	Choledocholithiasis without pancreatitis, (*n* = 60)
Age, years (%)					
< 40	518 (23.9)	365 (28.9)	116 (16.5)	12 (13.6)	3 (5.0)
40–59	865 (39.9)	540 (42.8)	246 (35.0)	28 (32.1)	27 (45.0)
≥ 60	788 (36.4)	357 (28.3)	341 (48.5)	51 (54.3)	30 (50.0)
Male:Female	1:2.7	1:4.7	1:1.4	1:1.2	1:1.9
American Society of Anaesthesiologists score (%)					
1	726 (33.5)	465 (36.8)	204 (29.0)	20 (24.7)	13 (21.7)
2	1229 (56.7)	709 (56.2)	305 (43.4)	56 (58.0)	35 (58.3)
3	201 (9.3)	83 (6.6)	91 (12.9)	15 (17.3)	10 (16.7)
4	10 (0.5)	5 (0.4)	3 (0.4)	0 (0)	2 (3.3)
Number of previous admissions (%)					
1	711 (34.2)	186 (14.7)	435 (61.9)	72 ()	36 (60.0)
2	133 (6.1)	16 (1.3)	96 (13.7)	13 ()	7 (11.7)
≥ 3	41 (1.9)	4 (0.3)	33 (4.7)	2 ()	1 (1.7)
Pre-operative ERCP	220 (10.2)	3 (0.2)	127 (18.1)	28 (28.4)	60 (100)
Pre-operative Cholecystostomy	36 (1.7)	0 (0.0)	36 (5.1)	0 (0)	0 (0)
Additional biliary disease					
Choledocholithiasis	211 (9.8)	–	133 (18.9)	18 (19.8)	60 (100)
Gallstone pancreatitis	174 (8.0)	–	83 (11.8)	91 (100)	0 (0)

*Including patients with biliary dyskinesia or gallbladder polyps

The rate of pre-operative MRCP was 29.9%. Thirty-one patients had a pre-operative cholecystostomy (1.7%), and 220 patients (10.2%) had a pre-operative ERCP. Following pre-operative ERCP, 8 patients (3.6%) had post-ERCP pancreatitis, 3 patients (1.4%) duodenal perforation, 1 patient (0.5%) haemorrhage requiring transfusion and 1 patient (0.5%) cholangitis. Of the above 13 patients with a complication related to ERCP, two patients suffering post-ERCP pancreatitis proceeded to a subtotal cholecystectomy (STC), one of which suffered a post-operative collection and the other a post-operative bile leak.

Overall, the rate of STC and/or conversion to open (CTO) cholecystectomy was 3.7%. In 19 patients (0.9%), a previous cholecystectomy attempt was abandoned before a second successful attempt was performed. The rate of intra-operative cholangiogram was 1.4% (31 patients), five of which showed a possible stone and three of these proceeded to ERCP. The rate of intra-operative drain insertion was 6.0%.

The rate of post-operative complication (Clavien–Dindo ≥ 2) was 4.8%. The rate of prolonged post-operative stay (≥ 3 days) was 6.6%, and the rate of re-admission was 6.0%.

Four patients (0.18%) had a bile duct injury, one of which was a complete transection of the common bile duct (0.05%). The mortality over the 100-day follow-up period was 0.04% (1/2166; one patient with gallbladder adenocarcinoma and lung metastasis).

### STC and CTO

There were 60 cases (2.8%) of subtotal cholecystectomy and 26 cases (1.2%) of conversion to open. Causes for STC included significant adhesions in cholecystohepatic triangle (36), intrahepatic gallbladder (7), cholecystoduodenal adhesions (5), gallbladder mass (2), Mirizzi syndrome (2), abnormal biliary anatomy (2), cholecystoduodenal fistula (2), cholecystocolonic adhesions (2) and cholecystocolonic fistula (1).

**Fig. 1:**
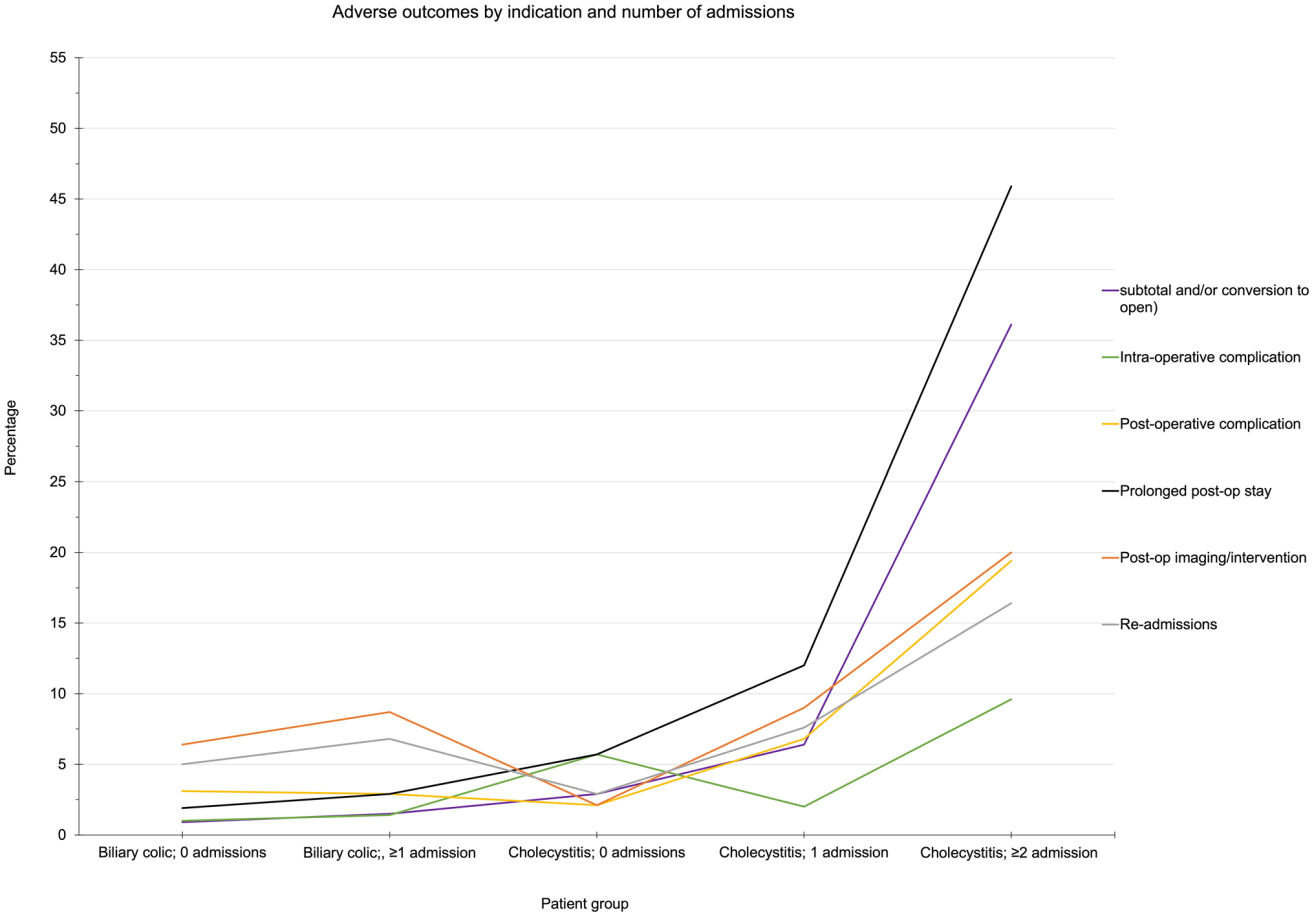
Adverse outcomes by indication (biliary colic and cholecystitis) and number of admissions

The rate of STC in the group of patients with biliary colic and no previous hospital admissions was 0.7% which was not significantly different than those with biliary colic and previous admissions (Table 2, Fig. 1). The rate of STC increased in the group with cholecystitis as the number of admissions with cholecystitis increased (*p* < 0.05). The rate of STC was highest (29.5%) in those with ≥ 2 admissions with cholecystitis.

**Table 2:** Outcomes by indication and number of admissions

Indication and number of admissions	Median operation length, (minutes)	Subtotal, (%)	Conversion to open, (%)	Intra-op complication, (%)	Post-op complication, (%)	Prolonged post-op stay, (%)	Post-op imaging, (%)	Post-op intervention, (%)	Re-admissions, (%)
All patients, (*n* = 2166)	72	62 (2.9)	26 (1.2)	40 (1.8)	104 (4.8)	144 (6.6)	175 (8.1)	57 (2.6)	132 (6.1)
Biliary colic									
No admissions, (*n* = 1056)	66	7 (0.7)	3 (0.3)	10 (1.0)	33 (3.1)	20 (1.9)	68 (6.4)	16 (1.5)	53 (5.0)
≥ 1 admissions, (*n* = 206)	70	2 (1.0)	0 (0)	3 (1.4)	6 (2.9)	6 (2.9)	18 (8.7)	3 (1.4)	14 (6.8)
Cholecystitis									
No admissions, (*n* = 140)	79	2 (1.4)	2 (1.4)	8 (5.7)	3 (2.1)	8 (5.7)	5 (3.5)	2 (1.4)	4 (2.9)
1 admission (*n* = 502)	85	28 (5.6)	9 (1.8)	10 (2.0)	34 (6.8)	60 (12.0)	48 (9.6)	21 (4.2)	38 (7.6)
≥ 2 admission (*n* = 61)	116	18 (29.5)	6 (9.8)	6 (9.8)	12 (19.6)	28 (45.9)	11 (18.0)	9 (14.8)	10 (16.4)
Gallstone pancreatitis, (*n* = 91)	72	2 (2.2)	2 (2.2)	1 (1.1)	6 (6.6)	10 (11.0)	9 (9.9)	2 (2.2)	5 (5.5)
Choledocholithiasis without pancreatitis, (*n* = 60)	74	3 (5.0)	4 (6.7)	2 (3.3)	6 (10.0)	10 (16.7)	10 (16.7)	4 (6.7)	6 (10)

Causes for CTO included adhesions in cholecystohepatic triangle (14), cholecystoduodenal adhesions (3), haemorrhage (2), adhesions from previous surgery (2), gallbladder mass (2), bile duct injury (1), abnormal biliary anatomy (1), cholecystocolonic fistula (1) and cholecystoduodenal fistula (1). Rates of conversion to open were higher in those with cholecystitis and more admissions versus those with biliary colic and no admissions (*p* < 0.05, Table 3).

**Table 3:** Relative risk of outcomes compared to biliary colic, no admissions group

Indication and number of admissions	Subtotal	Conversion to open	Intra-operative complication	Post-op complication	Prolonged post-op stay	Post-op imaging	Post-op intervention	Re-admissions
Biliary colic								
≥ 1 admission (*n* = 206)	1.4	0	1.4	0.9	1.5	1.4	0.9	1.4
Cholecystitis								
No admissions (*n* = 140)	2.0	4.7*	5.7***	0.7	3.0**	0.5	0.9	0.6
1 admission, (*n* = 502)	8.0***	6.0***	2.0	2.2***	6.3***	1.5*	2.8***	1.5*
≥ 2 admissions, (*n* = 61)	41.4***	32.0***	9.6***	6.3***	23.8***	2.8***	9.7***	3.2***
Gallstone pancreatitis (*n* = 91)	3.1	7.3**	1.1	2.1	5.8***	1.5	1.5	1.1
Choledocholithiasis without pancreatitis, (*n* = 60)	7.1***	22.3***	3.3	3.2**	8.8***	2.6**	4.5**	2.0

*Statistically significant difference from biliary colic, no admissions group (*p* < 0.05), **p < 0.01, ***p < 0.001

In the multivariate regression, factors associated with STC and/or CTO included age ≥ 60 years (OR 2.09;*p* = 0.002), cholecystitis (OR 3.55;*p* < 0.001), pre-op ERCP (OR 2.55;*p* = 0.001), 2 previous admissions (OR 2.32;*p* = 0.007) and ≥ 3 previous admissions (OR 4.89;*p* < 0.001), (Table 4).

**Table 4:** Multivariate logistic regression models—pre-operative factors associated with key adverse outcome measures

Outcome measure	Dependent variable	Odds Ratio	Std. Err.	*Z*	*p*-value	95% CI
Subtotal cholecystectomy and/or open cholecystectomy)	Age ≥ 60	2.09	0.51	3.03	0.002	1.30–3.38
	2 previous admissions	2.32	0.73	2.68	0.007	1.25–4.30
	≥ 3 previous admissions	4.89	2.09	3.72	< 0.001	2.12–11.29
	Cholecystitis	3.55	0.97	4.66	< 0.001	2.08–6.05
	Pre-op ERCP	2.55	0.70	3.42	0.001	1.49–4.36
Intra-operative complication	Age 40–60	5.46	4.10	2.26	0.024	1.25–23.76
	Age ≥ 60	5.62	4.21	2.30	0.021	1.29–24.42
	≥ 3 previous admissions	4.57	2.62	2.64	0.008	1.48–14.07
	Cholecystitis	2.56	0.87	2.76	0.006	1.31–4.98
Post-operative complication	ASA ≥ 3	1.70	0.50	1.82	0.049	1.02–3.02
	Cholecystitis	2.46	0.81	2.73	0.006	1.29–4.71
	Pre-op ERCP	2.33	0.62	3.22	< 0.001	1.39–3.92
Prolonged post-operative stay (≥ 3 days)	Male sex	1.61	0.31	2.47	0.013	1.10–2.36
	Age ≥ 60	1.67	0.25	3.47	< 0.001	1.25–2.24
	ASA 2	2.64	0.74	3.46	0.001	1.52–4.57
	ASA ≥ 3	4.46	1.49	4.47	< 0.001	2.31–8.59
	2 previous admissions	2.11	0.56	2.83	0.005	1.26–3.54
	≥ 3 previous admissions	3.23	1.31	2.89	0.004	1.46–7.14
	Cholecystitis	2.50	0.51	4.47	< 0.001	1.67–3.74
	Pre-operative cholecystostomy	3.02	1.17	2.85	0.004	1.41–6.45
	Pre-operative ERCP	2.54	0.56	4.24	< 0.001	1.65–3.91
Readmission	ASA ≥ 3	1.71	0.43	2.11	0.035	1.04–2.81
	≥ 2 previous admissions	2.31	0.66	2.92	0.003	1.32–4.04

### Intra-operative and post-operative complications

There was no significant difference in rates of complications in those with biliary colic as number of admissions increased; however, rates of intra-operative complications were higher in patients with cholecystitis (*p* < 0.05) (Table 2, Fig. 1). Furthermore, rates of post-operative complication were higher in the cholecystitis cohort as the number of admissions with cholecystitis increased (*p* < 0.05, Table 3). Rates of intra-operative and post-operative complications were highest in those with ≥ 2 admissions and with cholecystitis (9.6% and 19.4%, respectively).

In the multivariate regression, factors associated with intra-operative complications include age 40–60 (OR 5.46;*p* = 0.024), ≥ 60 years (OR 5.62;*p* = 0.021), cholecystitis (OR 2.56;*p* = 0.006) and ≥ 3 previous admissions (OR 4.57;*p* = 0.008). Variables associated with post-operative complications include ASA ≥ 3 (OR 1.70;*p* = 0.049), cholecystitis (OR 2.46;*p* = 0.006) and pre-operative ERCP (OR 2.33;*p* < 0.001) (Table 4).

The rates of both post-operative imaging and re-intervention were higher in the cholecystitis groups and increased as the number of admissions with cholecystitis increased (*p* < 0.05) (Table 2, Fig. 1). In the group with ≥ 2 admissions with cholecystitis, the rates of post-op imaging and re-intervention were 17.7% and 14.5%, respectively.

### Prolonged post-operative stay

The risk of prolonged post-operative stay increased with number of overall biliary related admissions and episodes of admissions with cholecystitis (*p* < 0.05; Table 2). The rate of prolonged post-operative stay was 45.2% in patients with ≥ 2 admissions with cholecystitis.

Factors associated with prolonged post-operative stay include male sex (OR 1.61;*p* = 0.013), age ≥ 60 (OR 1.67;*p* < 0.001), ASA 2 (OR 2.64;*p* = 0.001), ASA ≥ 3 (OR 4.46;*p* < 0.001), cholecystitis (OR 2.50;*p* < 0.001), pre-operative cholecystostomy (OR 3.02;*p* = 0.004), pre-operative ERCP (OR 2.54;*p* < 0.001), 2 previous admissions (OR 2.11;*p* = 0.005) and ≥ 3 previous admissions (OR 3.23;*p* = 0.004), (Table 4).

### Re-admissions

The re-admission rate was highest in the patients with ≥ 2 admissions with cholecystitis (16.4%) (Table 1). In the multivariate regression, factors associated with re-admission after ELLC include ASA ≥ 3 (OR1.71;*p* = 0.035) and ≥ 2 previous admissions (OR 2.31;*p* = 0.003) (Table 4).

## Discussion

This analysis demonstrates that patients undergoing LC represent a heterogenous group with considerable variation in outcomes. Modern practice in the United Kingdom places great emphasis on consent prior to interventions [8]. To ensure patients receive truly informed consent, the discussion and documentation of adverse outcomes should not be homogeneous process but instead should be guided by individual patient risk factors. This study allows for the identification of some of these risk factors. Similar studies have concentrated on risk factors for conversion to open surgery, looked at outcomes in cohorts including emergency patients or focused purely on selected groups such as elderly patients [1, 2, 4, 9–11]. The unique strength of this paper is that it considers a variety of generic outcomes in a large cohort of unselected patients scheduled to undergo ELLC.

The finding that a previous admission of cholecystitis is an independent risk factor for intra- and post-operative complications, prolonged stay and re-admission is not surprising and can result in a longer operation, the potential for a conversion or a subtotal cholecystectomy. It is noteworthy that repeated admission with cholecystitis increases the rates of these adverse outcomes.

It has previously been shown that severe cholecystitis is associated with post-operative complications following emergency operation [4]. Until this study, it was unclear how the risk of post-operative complications changes in the elective setting after episodes of cholecystitis. Our data demonstrate that the peri- and post-operative risk of a LC for cholecystitis persists once the period of active inflammation has settled.

Age has previously been described as a risk factor for poorer outcomes following biliary surgery [2]. Likewise, our finding that advanced ASA status is associated with prolonged post-operative stay is to be expected. These two factors often co-exist and one can suggest that surgeons discussing operative intervention with older, frail patients must highlight that the post-operative recovery may be protracted and not be the “day case procedure” often experienced by a younger, fitter cohort.

It has been commonly accepted that male sex is a factor associated with a difficult cholecystectomy. In the present study, male sex was associated with prolonged post-operative stay. Aside from this, this belief was not validated in our study and male sex alone once adjusted for other pre-operative factors was not positively associated with a difficult ELLC.

Cholecystostomy has been used more frequently during the COVID pandemic and has been described as “an effective and safe treatment thus acquiring an increased relevance” [12]. However, patients in this series who had undergone a prior cholecystostomy had a significantly longer post-operative stay. Cholecystostomy may be regarded as an appropriate intervention for those who are unfit for surgery at that time, but a proportion of these patients do subsequently undergo LC [13]. The results of this study would suggest that percutaneous gallbladder drainage must be used selectively and judiciously in those patients who may ultimately be surgical candidates.

Patients who have undergone pre-operative ERCP have an increased incidence of difficult cholecystectomy with complications and re-admission. It would be overly aspirational to suggest that the need for therapeutic ERCP can be dispensed with altogether. However, this is an important reminder that laparoscopic common bile duct exploration has a very acceptable safety profile and may often offer a cost effective solution for those patients with choledocholithiasis [14, 15]. Clearly, no ubiquitous solution exists for this common problem, but these data do hint that a modern biliary service should include the option of laparoscopic clearance of the common bile duct at the time of cholecystectomy for some patients.

In conclusion, our data demonstrate that patients with repeated biliary admission (particularly cholecystitis) ultimately face an increased risk of a difficult cholecystectomy with associated complications, prolonged post-operative stay and readmissions. These data provide robust evidence that individualised risk assessment, and consent is necessary before ELLC. Some risk factors are modifiable and efforts must be made to avoid patients having repeated admissions with biliary issues, particularly with cholecystitis. In this respect, the aims of improving surgical outcomes and resource utilisation are closely aligned.
